# In vitro efficacy of disinfectants against Cutibacterium acnes

**DOI:** 10.1099/jmm.0.002100

**Published:** 2025-11-20

**Authors:** Koyo Yoshihara, Shoji Seyama, Juri Koizumi, Hidemasa Nakaminami

**Affiliations:** 1 Department of Clinical Microbiology, School of Pharmacy, Tokyo University of Pharmacy and Life Sciences, 1432-1 Horinouchi, Hachioji, Tokyo 192-0392, Japan; 2 Laboratory of Microbiology and Immunology, Gifu Pharmaceutical University, 1-25-4 Gifu 501-1196, Japan

**Keywords:** acne vulgaris, *Cutibacterium acnes*, disinfectants, olanexidine, over the counter (OTC)

## Abstract

**Introduction.** The abnormal proliferation of *Cutibacterium acnes*, a human skin commensal bacterium, aggravates acne. Prompt and aggressive treatment of acne in its initial stages is essential because of the increased psychological distress experienced by patients. Over-the-counter medications containing disinfectants are sometimes used to treat acne because they are widely available, exhibit broad-spectrum activity and have a likelihood of causing resistance.

**Gap statement.** Limited information is available regarding the bactericidal effects of disinfectants against *C. acnes*.

**Aim.** In the present study, we comprehensively evaluated the efficacy of various disinfectants.

**Methodology.** *C. acnes* ATCC6919 and eight clinical isolates with different genotypes obtained from patients with acne were used. Benzethonium, chlorhexidine, resorcinol, homosulfamine, isopropyl methylphenol and olanexidine were used as disinfectants. The bactericidal activity of these disinfectants against *C. acnes* was assessed using a time–kill kinetic assay. Three independent experiments were conducted to ensure reproducibility of the results.

**Results.** Benzethonium, chlorhexidine, isopropyl methylphenol and olanexidine exhibited high efficacy. However, under *in vitro* acne conditions, with the addition of artificial sebum, the bactericidal efficacy of the disinfectants was significantly reduced. However, olanexidine retained bactericidal activity at concentrations as low as 0.2%, comparable with that at 1.5%.

**Conclusion.** This study showed that the disinfectants benzethonium, isopropyl methylphenol, chlorhexidine and olanexidine have high bactericidal activity against *C. acnes*. In particular, olanexidine demonstrated a strong bactericidal effect even under *in vitro* acne conditions. Further *in vivo* validation is required to determine whether olanexidine could be a new treatment option for managing acne.

## Introduction

*Cutibacterium acnes* and *Staphylococcus epidermidis*, which are components of the human skin microbiota, act as biological barriers to prevent the fixation and proliferation of pathogens, thus contributing to homeostasis [1]. *C. acnes* is particularly abundant in sebaceous areas of the skin, and its abnormal proliferation in occluded follicles induces inflammation, exacerbating acne vulgaris [2,3].

Acne is a chronic inflammatory skin disease commonly experienced during adolescence and is exacerbated by endocrine factors (such as androgen-induced sebum production), bacteriological factors and abnormal keratinization [4]. Few patients seek medical attention in its early stages because acne is often perceived as a ‘symbol of youth’ and is not fatal. However, acne frequently appears on the face and chest, leaving scars that significantly increase psychological distress of patients [5]. Therefore, aggressive early-stage treatment is crucial for improving quality of life. In addition, academics, companies and medical institutions involved in acne research have promoted public awareness campaigns regarding the importance of primary care.

Antimicrobial agents are used to reduce acne vulgaris in healthcare services provided by health insurance; antimicrobial agents are used to reduce the overgrowth of *C. acnes*. The Japanese guidelines for treating acne vulgaris recommend topical antimicrobial agents, such as clindamycin, nadifloxacin and ozenoxacin, as well as oral antimicrobial agents, such as doxycycline, minocycline and roxithromycin [6]. In addition to their antimicrobial activity, these antimicrobials have anti-inflammatory effects, thereby reducing the inflammation caused by acne [7]. However, there are several limitations to current acne treatments, including the emergence of antimicrobial-resistant *C. acnes* and the need for a prescription from a doctor. These issues highlight the need for alternative therapeutic strategies to eliminate prolonged administration of antibiotics. Adapalene and benzoyl peroxide are used to inhibit comedone formation. Cosmetic treatments, including laser and radiofrequency procedures, are also widely used [8,10]. However, many patients with acne use over-the-counter drugs available in drugstores and pharmacies without a prescription and at their own discretion [11,12].

In Japan, drugs for acne treatment include disinfectants such as quaternary ammonium salts (benzethonium), phenols (isopropyl methylphenol and resorcinol), biguanide (chlorhexidine) and sulphonamide (homosulfamine). Compared with conventional antimicrobial therapies, disinfectants used in over the counter (OTC) drugs may offer several advantages, including lower cost, broad-spectrum activity and a reduced likelihood of promoting antimicrobial resistance. Many of these agents have been approved for cutaneous disinfection, suggesting potential safety benefits. These agents exhibit bactericidal activity by disrupting bacterial cell membranes, proteins and enzymes [13]. In addition, olanexidine gluconate, a novel biguanide disinfectant, was introduced in 2015 and is used for surgical site disinfection. Olanexidine interacts with bacterial surface molecules such as lipopolysaccharides and lipoteichoic acid, disrupting cell membranes and denaturing proteins, thereby causing irreversible leakage of intracellular components and exhibiting bactericidal effects [14]. This mechanism is slightly different from that of chlorhexidine. Additionally, olanexidine demonstrates strong broad-spectrum antibacterial activity against antimicrobial-resistant bacteria and immediate efficacy. The minimum bactericidal concentration of chlorhexidine against *C. acnes* is higher than commercially available concentrations, whereas that of olanexidine is sufficiently low [14]. However, information regarding the efficacy of these disinfectants against *C. acnes* is limited and requires further evaluation. Acne pyocysts contain abundant sebum, such as squalene, triglycerides, fatty acids and wax esters [15]. Evaluation of the bactericidal effect of topical drugs against *C. acnes* in acne pyocysts under these conditions involves three factors: the bactericidal potency of the drug, its efficacy in the presence of sebum and its pyocyst permeability. This study evaluated the bactericidal activity of six disinfectants, representing different chemical classes and mechanisms of action, against *C. acnes in vitro*. Disinfectants were selected from skin antiseptics and OTC drugs commonly used in Japan.

## Methods

### Bacterial strains, cells and culture conditions

*C. acnes* ATCC6919 and a strain derived from a patient with acne were used as quality control strains in this study [16]. Two *C. acnes* strains from each clade were used. These strains were selected from clinical isolates of patients with acne in our stock. Single-locus sequence typing (SLST) clade A, C and F strains are commonly isolated from patients with acne, whereas clade K strains are often found in medical devices as well as in blood and soft tissue samples. Eight clinical isolates with different genotypes, classified by SLST, were used: clade A, two isolates; clade C, two isolates; clade F, two isolates; and clade K, two isolates [17]. *C. acnes* strains were cultured on modified Gifu anaerobic medium (GAM) agar (Nissui Pharmaceutical, Tokyo, Japan) at 37 °C for 72 h under anaerobic conditions. Human epidermal keratinocyte cell line [human adult low calcium temperature (HaCaT) cells, which are human monolayer epidermal cells] was cultured in Keratinocyte Cell Basal Medium (KBM)-Gold™ (500 ml medium supplemented with: hydrocortisone 0.5 ml, transferrin 0.5 ml, epinephrine 0.25 ml, gentamicin sulfate-amphotericin (GA-1000) 0.5 ml, bovine pituitary extract 2.0 ml, insulin 0.5 ml and recombinant human epidermal growth factor 0.5 ml; Lonza, Basal, Switzerland) with penicillin–streptomycin solution (×100; FUJIFILM Wako Pure Chemical, Tokyo, Japan) at 37 °C under 5% carbon dioxide (CO_2_).

### Disinfectants

Benzethonium chloride, isopropyl methylphenol, resorcinol (FUJIFILM Wako Pure Chemical), chlorhexidine gluconate (Sigma-Aldrich, Tokyo, Japan) and homosulfamine (Combi-Blocks, CA, USA) were used as disinfectants. Olanexidine gluconate was provided by the Otsuka Pharmaceutical Factory. The test concentrations were selected based on practical usage. Benzethonium chloride (0.05%), chlorhexidine (0.2%), homosulfamine (0.3%) and olanexidine (0.2% and 1.5%) were dissolved in sterile saline solution. Isopropyl methylphenol (1 and 0.3%) and resorcinol (2%) were dissolved in 10% dimethyl sulfoxide (DMSO).

### Bactericidal activity of disinfectants

The bactericidal activities of the disinfectants were evaluated using a time–kill kinetic assay. As the test bacterial solution, *C. acnes* strains were inoculated into sterilized saline to an optical density of 1.0 at 600 nm. The bacteria were inoculated into saline or 10% DMSO (FUJIFILM Wako Pure Chemical) at 10^6^ c.f.u. ml^−1^. Bacterial growth was evaluated after 1 and 5 min by counting the number of viable cells on GAM agar plates. The disinfectants were neutralized using a solution confirmed to completely inactivate them without affecting bacterial viability. The neutralizing solution contained 10% TWEEN 80 (SigmaAldrich), 1.17% lecithin (The Nisshin OilliO Group, Ltd., Tokyo, Japan), 0.5% sodium thiosulphate (FUJIFILM Wako Pure Chemical), 0.04% potassium dihydrogen phosphate (FUJIFILM Wako Pure Chemical), 0.1% (v/v) Triton X-100 (FUJIFILM Wako Pure Chemical), 1.01% disodium phosphate (FUJIFILM Wako Pure Chemical) and 1% Tamol NN8906 (BASF Japan, Tokyo, Japan), as previously described [18]. The number of proliferating colonies was counted after incubation on GAM agar at 37 °C for 72 h under anaerobic conditions.Artificial sebum was prepared following Wertz *et al*. (2009) and Nakase *et al*. (2011) [19,20]. It contained 10% artificial sebum, consisting of 12.5% (w/w) squalene, 25% (w/w) jojoba oil, 45% (w/w) triolein and 17% (w/w) oleic acid (*in vitro* acne model), all from FUJIFILM Wako Pure Chemical. These proportions replicate human sebum and recreate the microenvironment of acne-affected hair follicles. As a control, *C. acnes* cells were cultured in the same medium without sebum supplementation. These experiments were performed at least three times on independent occasions, and the results are shown as the mean ± sd.

### Permeability of disinfectants into HaCaT cell

The permeability of disinfectants was determined using HaCaT cells layered on Transwell inserts [24-well, polyethylene terephthalate (PET) 0.4 µm; Sarstedt AG and Co. KG, Nümbrecht, Germany] to calculate drug permeability to the lower layer. The control condition was inoculated without HaCaT cells, and the disc method was used to measure the diameter of the inhibition circle and to generate a calibration curve. Transwells were placed in 24-well plates, and HaCaT cells were inoculated at 1×10^5^ cells/well and cultured at 37 °C for 24 h under 5% CO_2_. The medium was then removed, and benzethonium, chlorhexidine and olanexidine were each dissolved in Keratinocyte Growth Medium (KGM) at 0.05%, 0.2% and 1.5%, respectively. A total of 100 µl of each solution was added dropwise to the wells and incubated at 37 °C for 1 h under 5% CO_2_. Next, 6 mm paper discs (Advantec, Tokyo, Japan) were placed onto GAM agar plates containing *C. acnes*. Subsequently, 80 µl of the lower layer was added to each disc. The plates were then incubated at 37 °C for 48 h under anaerobic conditions. The diameter of the inhibition zone was measured after incubation. The estimated concentration that penetrated the pyocysts was calculated using the regression constant and coefficient derived from the inhibition zone and the calibration curves generated under cell inoculation conditions. The calculated concentrations were used to evaluate the bactericidal effects in an *in vitro* acne model using a time–kill kinetic assay. The results are shown as the mean ± sd, derived from three independent experiments.

### Statistical analysis

All experiments were performed independently in triplicate (*n*=3), and the results are presented as the mean ± sd, unless otherwise indicated. Colony counts (c.f.u. ml^−1^) were log_₁₀_ transformed before analysis to stabilize variance. Data normality was assessed using the Shapiro–Wilk test, and homogeneity of variances was tested using Levene’s test. For comparisons between two groups, a two-tailed Student’s t-test was applied to normally distributed data. Exact *P*-values are reported in the text or figure legends, and *P*<0.05 was considered statistically significant.

## Results

### Bactericidal activity of disinfectants

**Fig. 1.**
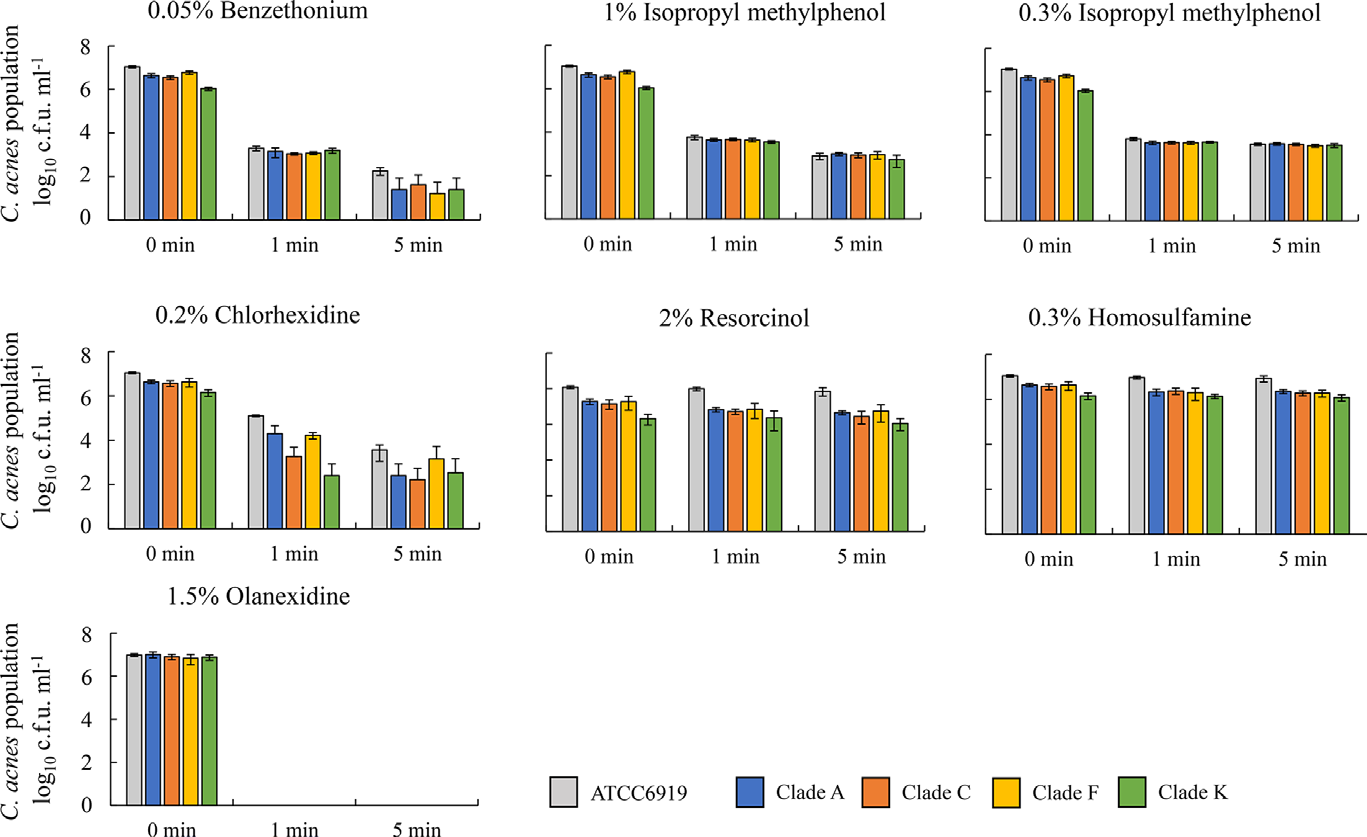
Bactericidal effect of disinfectants against *C. acnes* strains, including clinical isolates, using an *in vitro* skin model. The bactericidal effects were evaluated using a time–kill kinetic assay. *C. acnes* strain ATCC6919 and clinical isolates belonging to SLST clades A, C, F and K were used. Benzethonium, isopropyl methylphenol, chlorhexidine and olanexidine exhibited strong bactericidal effects.

The bactericidal activity of the disinfectants against *C. acnes* in different SLST clades was evaluated. The 0.05% benzethonium, 1% and 0.3% isopropyl methylphenol, 0.2% chlorhexidine and 1.5% olanexidine exhibited strong bactericidal activity (Fig. 1).In particular, olanexidine reduced the bacterial counts to the detection limit within 1 min. Olanexidine, benzethonium, chlorhexidine and isopropyl methylphenol showed greater bactericidal activity against *C. acnes* than 2% resorcinol and 0.3% homosulfamine. No differences in bactericidal activity were observed across the different *C. acnes* SLST clades for any of the tested agents.

### Influence of sebum on bactericidal activity of disinfectants

**Fig. 2.**
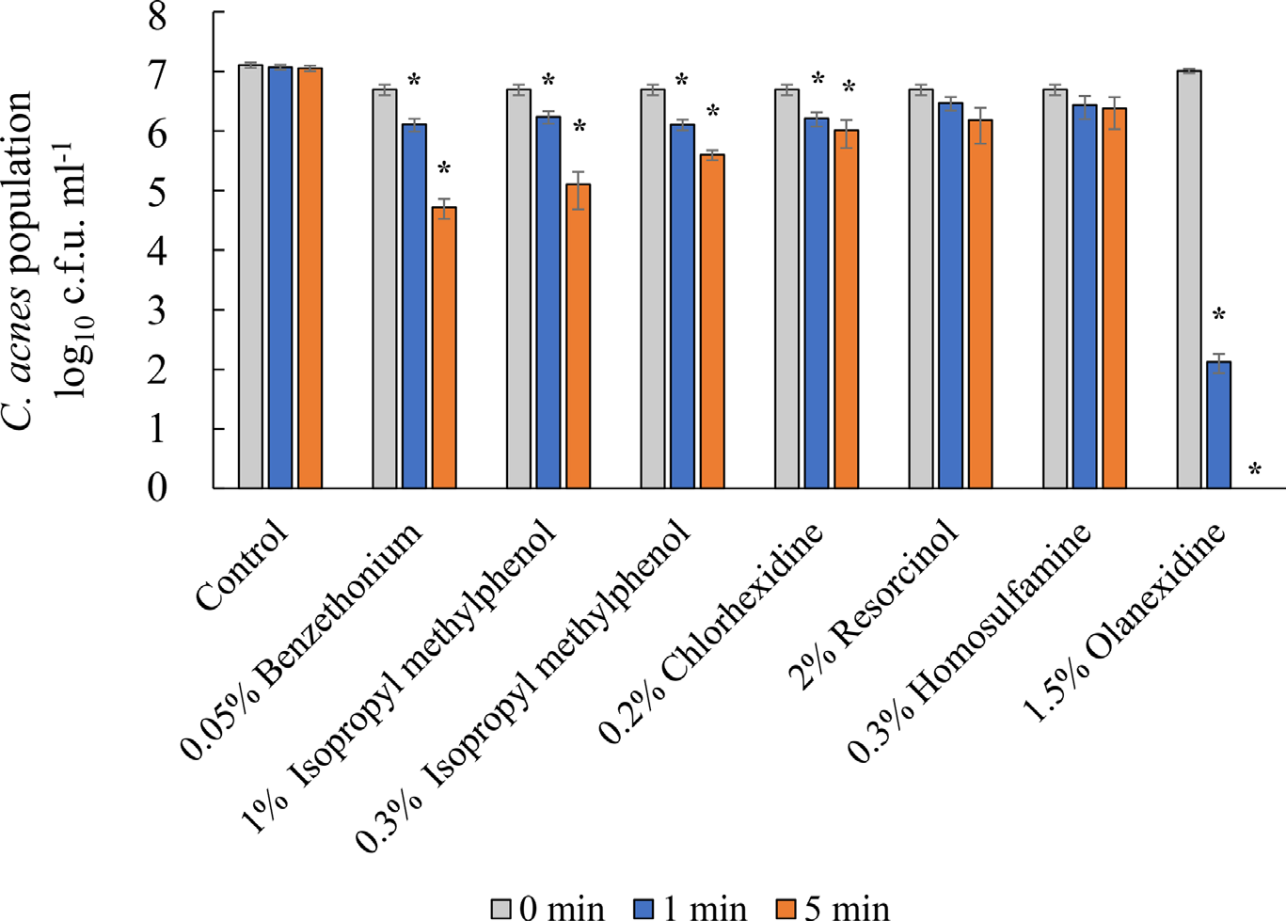
Comparison of bactericidal effects against *C. acnes* between the *in vitro* skin model and the *in vitro* acne model. Bactericidal effects against *C. acnes* ATCC6919 were evaluated using a time–kill kinetic assay. In the *in vitro* acne model, *C. acnes* was suspended in saline containing 10% artificial sebum. In the *in vitro* acne model, the bactericidal effects of benzethonium, isopropyl methylphenol and chlorhexidine were reduced, whereas those of olanexidine were not. Asterisks indicate significant differences versus 0 min for each group using Student’s t-test (*P*<0.05).

To clarify the bactericidal activity in acne pustules, the efficacy of various agents against the *C. acnes* ATCC6919 strain was evaluated using an *in vitro* acne model with the addition of artificial sebum (Fig. 2). The results indicated that, after 5 min, benzethonium showed reduced bactericidal activity, with bacterial counts ranging from below the detection limit to 5.3×10^4^ c.f.u. ml^−1^. Similarly, 1% isopropyl methylphenol resulted in bacterial counts between 5.8×10^3^ and 1.3×10^5^ c.f.u. ml^−1^, while 0.3% isopropyl methylphenol showed reduced bactericidal activity, with counts ranging from below the detection limit to 4.0×10^5^ c.f.u. ml^−1^. Chlorhexidine exhibited decreased bactericidal activity, with counts ranging from 3.6×10^3^ to 1.1×10^6^ c.f.u. ml^−1^. By contrast, olanexidine consistently demonstrated strong bactericidal activity in the *in vitro* acne model, maintaining bacterial counts below the detection limit after 5 min.

### Bactericidal effect of olanexidine in low concentration

**Fig. 3.**
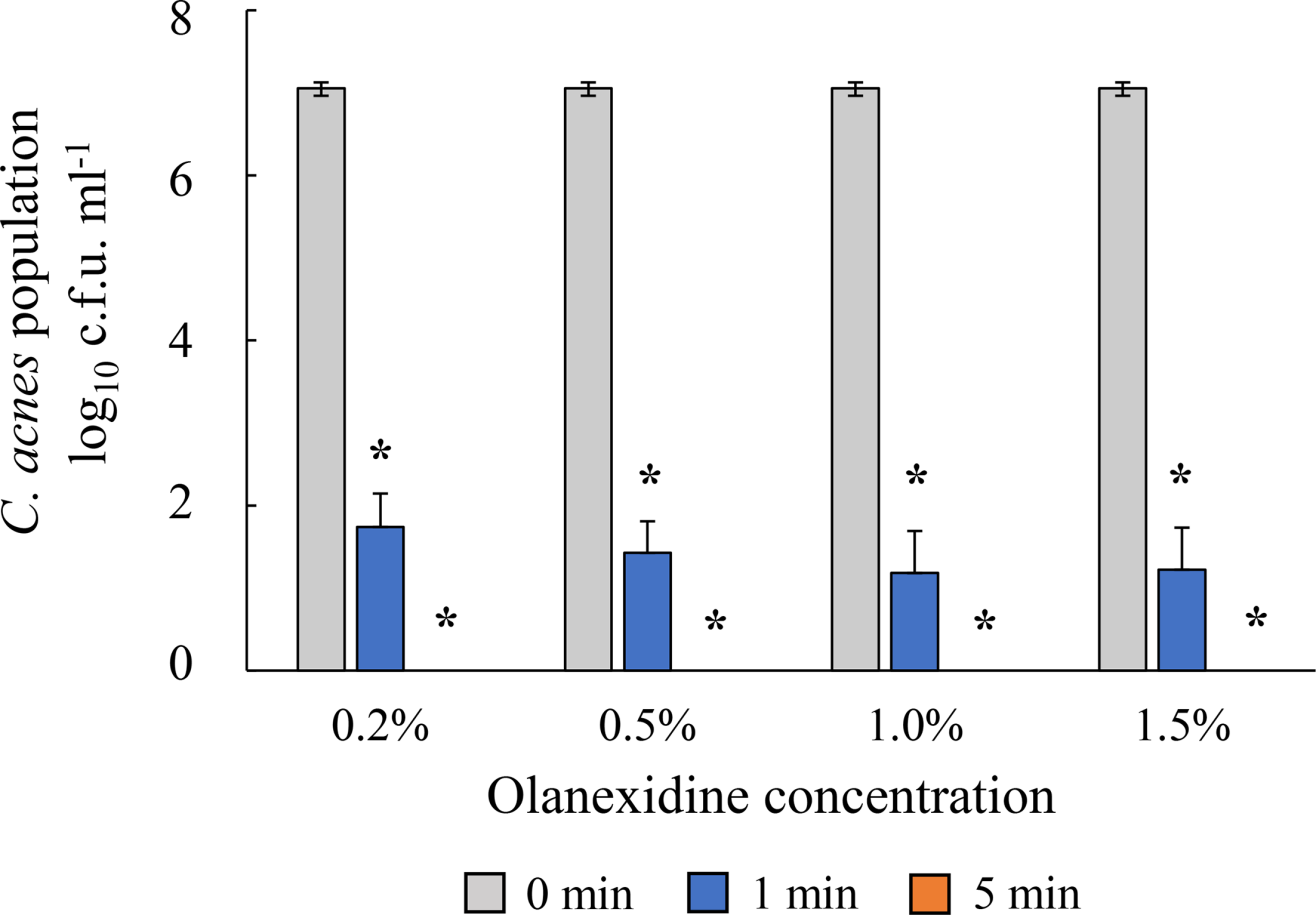
Bactericidal effect of olanexidine at low concentrations. The bactericidal effects of olanexidine were evaluated using a time–kill kinetic assay. Concentrations of 0.2%, 0.5%, 1.0% and 1.5% of olanexidine were used. The bactericidal effect of olanexidine against *C. acnes* was independent of concentration. Asterisks indicate significant differences versus 0 min for each group using Student’s t-test (*P*<0.05).

Because the concentration of 1.5% olanexidine, which is commonly used for surgical site disinfection, is higher than the concentrations of other disinfectants effective against *C. acnes*, we tested its bactericidal activity at lower concentrations (Fig. 3). The results showed that bactericidal activity was maintained across all tested concentrations in the *in vitro* acne model (Fig. 3). In addition, no significant difference was observed in bacterial reduction between the 0.2% and 1.5% concentrations after 1 min exposure (*P*=0.16).

### Permeability of disinfectants into HaCaT cells

**Fig. 4.**
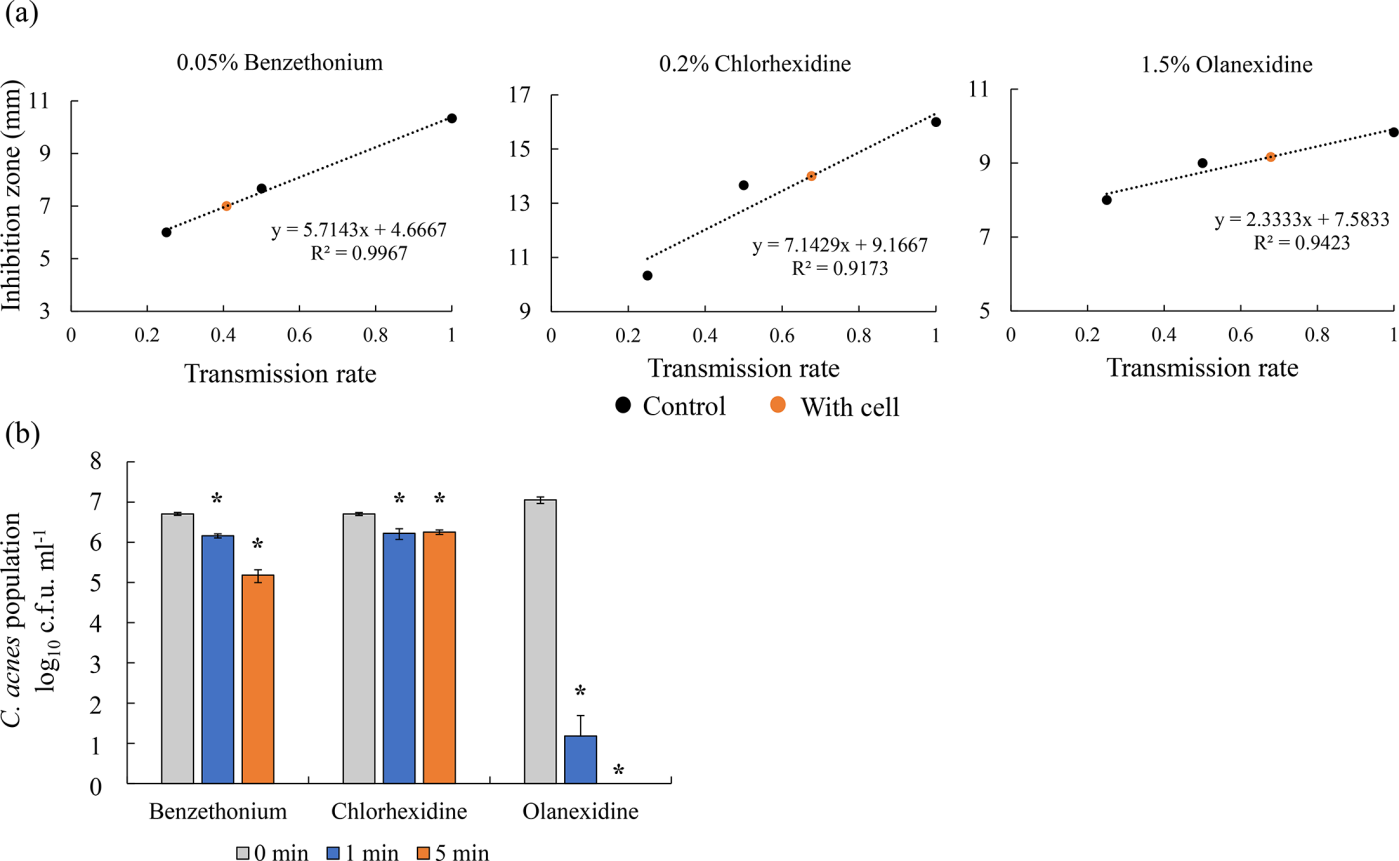
Permeability of disinfectants into HaCaT cells and bactericidal effects at transmitted concentrations. (**a**) Benzethonium, chlorhexidine and olanexidine, which showed strong bactericidal effects in the time–kill assay, were used. Permeability of disinfectants was calculated by seeding HaCaT cells on a Transwell insert. The inhibition zone was measured by using the disc method. A calibration curve was created based on the inhibition zone and concentration multiplier. Permeability to HaCaT cells was calculated from the regression constants and regression coefficients of the inhibition zone and the calibration curve. The permeabilities of benzethonium, chlorhexidine and olanexidine were 40.8%, 67.7% and 67.9%, respectively. (**b**) Bactericidal effects were evaluated using a time–kill kinetic assay in the *in vitro* acne model. At the transmitted concentrations of benzethonium, chlorhexidine and olanexidine, the number of *C. acnes* significantly decreased. Asterisks show significant differences versus 0 min for each group using Student’s t-test (*P*<0.05).

This assay evaluates the bactericidal effects of disinfectants at concentrations capable of penetrating the HaCaT cell layer, as well as membrane permeability. The permeability of 0.05% benzethonium, 0.2% chlorhexidine and 1.5% olanexidine, which showed high bactericidal activity in the *in vitro* acne model, was assessed in HaCaT epidermal cells (Fig. 4a). The results showed that the permeabilities were 40.8% for benzethonium, 67.7% for chlorhexidine and 67.9% for olanexidine. The bactericidal effect of these permeated concentrations was then evaluated in the *in vitro* acne model using *C. acnes* ATCC6919, and all agents demonstrated a significant reduction in the number of residual bacteria, although their activity was somewhat attenuated (Fig. 4b). Notably, olanexidine exhibited the strongest bactericidal activity among the tested agents.

## Discussion

Patients with acne who consult dermatologists are often treated with antimicrobials and other drugs to inhibit the growth of * C. acnes*. Many patients do not visit doctors but use disinfectants available at drugstores and pharmacies at their discretion [11,12]. In this study, we focused on disinfectants used for acne treatment and assessed their bactericidal effects against *C. acnes*.

The agents tested, including 0.05% benzethonium, 1% and 0.3% isopropyl methylphenol, 0.2% chlorhexidine and 1.5% olanexidine, showed strong bactericidal effects. By contrast, both resorcinol and homosulfamine were found to be less effective. Although resorcinol, a phenol-based compound, and homosulfamine, a sulphonamide, are used for their antibacterial properties, their primary mechanism of action may involve skin exfoliation rather than direct bactericidal action, similar to that of benzoyl peroxide and adapalene [13]. Therefore, their direct bactericidal effects on *C. acnes* are limited. In SLST-based genotyping of * C. acnes,* clade A strains are frequently isolated from the skin of healthy adults, clade C strains from European and American patients with acne, and clade F strains from Japanese patients with acne, with antimicrobial susceptibility varying by clade [16,21, 22]. Clade K strains are also thought to contribute to the maintenance of skin health as *C. acnes* subsp. *defendens* [23]. In this study, no differences were observed in the bactericidal effects of the various *C. acnes* SLST clades for the agents tested. Therefore, these five bactericidal agents may be effective against many *C. acnes* strains, regardless of the genotype.

Sebum production is higher in patients with acne than in healthy individuals [20]. Although benzethonium, chlorhexidine and isopropyl methylphenol showed strong bactericidal effects in the *in vitro* skin model under low-nutrient conditions without sebum, these effects were reduced in the presence of artificial sebum, suggesting that their bactericidal activity may decrease in actual acne lesions. By contrast, chlorhexidine, olanexidine and biguanide retained their bactericidal effects in the *in vitro* acne model. Typically, for surgical disinfection, a 1.5% concentration is used, which is much higher than the concentrations found in drugs for acne treatments. The bactericidal effect of olanexidine was evaluated at lower concentrations. As a result, no difference in bactericidal effect was observed between the lower concentrations and 1.5%, indicating that even at reduced concentrations, the bactericidal effect was sufficient. This plateau effect was probably caused by the high membrane affinity and rapid bactericidal activity of olanexidine. Once the agent reaches a sufficient concentration to disrupt bacterial membranes, further increasing the concentration may not enhance its bactericidal efficacy. Olanexidine may have a higher affinity for bacterial cell membranes than chlorhexidine and is less affected by organic matter [24]. Therefore, unlike chlorhexidine, olanexidine may maintain a strong bactericidal effect even in the presence of sebum. Thus, olanexidine may be a candidate for a new therapeutic agent for the treatment of acne. However, it has been reported that olanexidine can cause delayed-onset dermatitis and other skin symptoms in some patients [25,26]. This may be due to its prolonged presence on the surface of the skin layers. Therefore, olanexidine should be used with caution due to the potential risk of dermatitis. Compared with conventional treatments, such as benzoyl peroxide or topical antibiotics, disinfectants such as olanexidine may offer faster bactericidal activity and a lower risk of developing resistance. However, further direct comparative clinical studies are required to determine their efficacies and safety profiles.

In this study, the permeabilities of benzethonium, chlorhexidine and olanexidine into HaCaT cells were 40.8%, 67.7% and 67.9%, respectively. In addition, these agents significantly reduced bacterial counts at concentrations that penetrated HaCaT cells, with olanexidine being particularly effective.

This study has several limitations. First, the number of clinical isolates analysed within each clade was limited, which may reduce the generalizability of the findings. Because the comparison among SLST clades was not the primary focus of this study, no statistical analyses were performed for clade-related differences. Given the small number of isolates per clade (*n*=2), the statistical power was limited, and the results were interpreted descriptively. Second, while the *in vitro* model incorporating artificial sebum enabled controlled evaluation of drug efficacy under sebum-rich conditions, it does not fully replicate the complexity of the *in vivo* environment. These include host immune response, skin architecture and microbial diversity. Third, the exposure durations tested were relatively short and may not accurately reflect real-world application times. Therefore, future studies should include larger sample sizes, use *in vivo* models and assess longer exposure periods to enhance clinical relevance of the results. Although this study demonstrated the bactericidal activity of the disinfectants under controlled *in vitro* conditions, *in vitro* efficacy does not necessarily predict clinical outcomes. Real-world effectiveness can be significantly influenced by factors such as host immune response, skin barrier function and patient-specific variables.

In conclusion, this study showed that drugs containing benzethonium, isopropyl methylphenol and chlorhexidine have bactericidal activity against *C. acnes*, although their effectiveness may be diminished in regard to real acne pyocysts. By contrast, olanexidine retained its bactericidal activity in the presence of sebum and remained effective even at low concentrations. Our findings suggest that olanexidine can help prevent acne from worsening. However, further *in vivo* and clinical studies are required to confirm its efficacy and safety.

## Abbreviations

DMSO: dimethyl sulfoxide
GAM: Gifu anaerobic medium
OTC: over the counter
SLST: single-locus sequence typing
